# Gene Expression in Pre-MBT Embryos and Activation of Maternally-Inherited Program of Apoptosis to be Executed at around MBT as a Fail-Safe Mechanism in *Xenopus* Early Embryogenesis

**DOI:** 10.4137/grsb.s579

**Published:** 2008-05-29

**Authors:** Koichiro Shiokawa, Mai Aso, Takeshi Kondo, Hiroaki Uchiyama, Shinsaku Kuroyanagi, Jun-Ichi Takai, Senji Takahashi, Masayuki Kajitani, Chikara Kaito, Kazuhisa Sekimizu, Eiji Takayama, Kazuei Igarashi, Hiroshi Hara

**Affiliations:** 1 Department of Biosciences, School of Science and Engineering, Teikyo University; 1-1 Toyosatodai, Utsunomiya, Tochigi Prefecture 320-8551, Japan; 2 Graduate School of Pharmaceutical Sciences, University of Tokyo, Bunkyo-ku, Tokyo 113-0033, Japan; 3 Department of Parasitology and Immunology, National Defense Medical College, 3-2 Namiki, Tokorozawa, Saitama 359-8513, Japan; 4 Graduate School of Pharmaceutical Sciences, Chiba University, 1-8-1 Inohana, Chuo-ku, Chiba 263-8522, Japan; 5 Medical Research Laboratories, Taisho Pharmaceutical Company Ltd., Yoshino-cho 1-403, Kita-ku, Saitama-shi 330-0031, Japan

**Keywords:** *Xenopus laevis* embryos, overexpression of SAMDC, maternal program of apoptosis, caspases, p53, pre-MBT transcription, polyamines, rRNA synthesis

## Abstract

S-adenosylmethionine decarboxylase (SAMDC) is an enzyme which converts S-adenosylmethione (SAM), a methyl donor, to decarboxylated SAM (dcSAM), an aminopropyl donor for polyamine biosynthesis. In our studies on gene expression control in *Xenopus* early embryogenesis, we cloned the mRNA for *Xenopus* SAMDC, and overexpressed the enzyme by microinjecting its mRNA into *Xenopus* fertilized eggs. In the mRNA-injected embryos, the level of SAMDC was enormously increased, the SAM was exhausted, and protein synthesis was greatly inhibited, but cellular polyamine content did not change appreciably. SAMDC-overexpressed embryos cleaved and developed normally up to the early blastula stage, but at the midblastula stage, or the stage of midblastula transition (MBT), all the embryos were dissociated into cells, and destroyed due to execution of apoptosis. During cleavage SAMDC-overexpressed embryos transcribed caspase-8 gene, and this was followed by activation of caspase-9. When we overexpressed p53 mRNA in fertilized eggs, similar apoptosis took place at MBT, but in this case, transcription of caspase-8 did not occur, however activation of caspase-9 took place. Apoptosis induced by SAMDC-overexpression was completely suppressed by Bcl-2, whereas apoptosis induced by p53 overexpression or treatments with other toxic agents was only partially rescued. When we injected SAMDC mRNA into only one blastomere of 8- to 32-celled embryos, descendant cells of the mRNA-injected blastomere were segregated into the blastocoel and underwent apoptosis within the blastocoel, although such embryos continued to develop and became tadpoles with various extents of anomaly, reflecting the developmental fate of the eliminated cells. Thus, embryonic cells appear to check themselves at MBT and if physiologically severely-damaged cells occur, they are eliminated from the embryo by activation and execution of the maternally-inherited program of apoptosis. We assume that the apoptosis executed at MBT is a “fail-safe” mechanism of early development to save the embryo from accidental damages that take place during cleavage.

## Introduction

In *Xenopus* early embryogenesis, fertilized eggs go through the first cleavage following G_1_, S, G_2_, and M phases, but from the second cell cycle, cleavage proceeds rapidly without G_1_ and G_2_ phases until the end of the 12th cleavage (Heasman, 2006). During the cleavage, maternal mRNAs are actively translated (Woodland, 1974; Richter et al. 1982), but transcription of nuclear genes is not quite active (Brown and Littna, 1964, 1966; Shiokawa and Yamana, 1965, 1967; Shiokawa et al. 1981a, b). Therefore, maternal proteins and proteins translated from maternal mRNAs are believed to play key roles to drive the early phase of development (Davidson, 1976; Heasman, 2006). After the 12 rounds of cell cycles, embryos reach the midblastula stage, when G_1_ and G_2_ phases reappear (Graham and Morgan, 1966; Heasman, 2006) and embryos enter the phase of active formative movements (Gurdon, 1988, 2006).

During the transition from the early to late blastula stage, various changes in cellular activities take place. First, cleavage becomes asynchronous (Signoret and Lefresne, 1973; Newport and Kirschner, 1982), and cells acquire motility (Newport and Kirschner, 1982), especially in the future dorsal animal region (Minoura et al. 1995). Along with these changes, cell cycles shift from checkpoint-unregulated ones to checkpoint-regulated ones (Wroble and Sible, 2005), and in this remodeling process checkpoint kinases such as Chk2/Cds1 and Xchk1 play key roles (Wroble and Sible, 2005; Carter and Sible, 2003). Also, transcription from zygotic nuclear genes, including tRNA genes (Brown and Littna, 1964, 1966; Shiokawa and Yamana, 1967; Shiokawa et al. 1981a, b; Yang et al. 2002; Yasuda and Schweger, 1992; Shiokawa et al. 1994; Nakakura et al. 1987; Shiokawa et al. 1989) and exogenously-introduced genes like bacterial CAT (chloramphenicol acetyltransferase) genes (Etkin and Balcells, 1985; Shiokawa et al. 1990) is greatly activated on a “per-embryo” basis, but not necessarily on a “per-cell” basis, since there is low but significant transcription of both endogenous (Nakakura et al. 1987; Shiokawa et al. 1989; Shiokawa et al. 1994; Yang et al. 2002) and exogenously-introduced (Shiokawa et al. 1990) genes in preblastular embryos which are composed of relatively small number of cells. In addition, transcription of rRNA genes which was believed to start at gastrulation (Brown and Littna, 1964; Shiokawa and Yamana, 1967) was found to start at the late blastula stage based on the determination of newly formed rRNA-specific 2′-O-methylation (Shiokawa et al. 1981a, b). Thus, rRNA synthesis is undetectable (less than 0.08 ng/embryo/hr) at early blastula stage, but starts at the late blastula stage at a high rate of ca. 1.5 ng/embryo/hr (Shiokawa et al. 1981a, b), and formation of definitive nucleoli as a reliable cytological manifestation of rRNA synthesis (Gurdon and Brown, 1965), also starts from this stage (Nakahashi and Yamana, 1976). Transcription of exogenously-introduced rRNA genes (pXlr101A and its derivatives) also starts at the late blastula stage (Busby and Reeder, 1983).

These changes in various cellular activities during blastula stage were collectively called midblastula transition (MBT) in *Xenopus* (Signoret and Lefresne, 1973; Newport and Kirschner, 1982; Yasuda and Schweger, 1992; Shiokawa et al. 1994; Shiokawa et al. 1989; Andéol, 1994). Similar changes have been reported to occur also in zebrafish (Kane and Kimmel, 1993). Thus, it is only after MBT that extensive cellular movements (Nagel et al. 2004; Ninomiya et al. 2004; Shook et al. 2004) followed by various cell-to-cell interactions that include mesodermal and neural inductions take place (Gurdon, 1988; Heasman, 2006).

We have been studying mechanisms that control the transition from cleavage stage to the stage of extensive morphogenesis in *Xenopus* development. As one of our approaches to this goal, we analyzed polyamine metabolism in *Xenopus* early embryogenesis. In the course of the studies, we unexpectedly discovered the occurrence of cellular device which may be called the maternally-inherited program of apoptosis, or the apoptosis program main components of which already occur in unfertilized eggs. This apoptosis program appears to be triggered during cleavage stage or early blastula stage and is executed at MBT, and seems to be a mechanism which has physiological significance different from that of the apoptosis or the programmed cell death observed in brain segmentation (Shinga et al. 2001) or tail regression (Nishikawa et al. 1989; Tata et al. 1991; Wang and Brown, 1993; Shi et al. 1996). In the following, we summarize studies performed in our laboratory and in other laboratories in relation to the apoptosis program which is uniquely set in operation as early as the blastula stage in *Xenopus* embryogenesis.

## Discovery of Massive Cell Dissociation in SAMDC-Overexpressed *Xenopus* Blastulae

In *Xenopus* early embryos, but not in *Xenopus* oocytes and adult-type cells, spermine content is extremely low, although putrescine and spermidine content is relatively high (Shinga et al. 1996). Generally speaking, high spermine content is characteristic to eukaryotic cells and extremely low spermine content is characteristic to prokaryotic cells (Davis et al. 1992; Guirard and Snell, 1964), and in this sense polyamine content of *Xenopus* early embryos is like that of prokaryotes (Shinga et al. 1996). We, therefore, planned to artificially increase the level of spermine in early *Xenopus* embryos, since such an approach may provide a clue to elucidate the function of polyamines in *Xenopus* early embryogenesis. We cloned cDNA of SAMDC and overexpressed this enzyme in early *Xenopus* embryos by microinjecting its mRNA into fertilized eggs, since the overexpression was expected to provide embryos with an elevated level of dcSAM, an aminopropyl donor necessary to convert putrescine to spermidine and spermidine to spermine.

**Figure 1:**
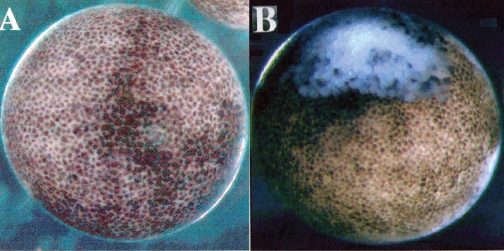
Induction of apoptosis by injection of *Xenopus* SAMDC mRNA. (A) A control embryo injected with distilled water. (B) A fertilized egg injected with *Xenopus* SAMDC mRNA (100 pg), and cultured in a slightly hypertonic 1 X Steinberg’s solution in order to protect dissociated cells from osmotic shock. White cells are dissociated cells. These cells appear in the region where mRNA was injected. Embryos were filmed at early gastrula stage. From Takayama et al. (2004).

In the mRNA-injected embryos, the activity of SAMDC increased over 400-fold (Shinga et al. 1996). Contrary to our expectation, however, the SAMDC overexpression exerted little influence on the polyamine composition within the embryo (Shinga et al. 1996), probably because we did not simultaneously overexpress spermine synthase, an enzyme necessary to transfer the aminopropyl residue to spermidine to form spermine (Pegg, 1986). SAMDC-overexpressed embryos cleaved and developed normally, and there was no difference in the time course and pattern of cleavage between the control and SAMDC-overexpressed embryos until the early blastula stage (Shibata et al. 1998). At the midblastula stage or at MBT, however, massive cell dissociation took place suddenly in SAMDC-overexpressed embryos (Fig. 1). We performed several control experiments, with the finding that SAMDC mRNA without a cap structure and mRNAs for other proteins such as β-galactosidase, type IIA activin receptor (Kondo et al. 1991), nrp-1 (Richter et al. 1990) and eIF4E (Wakiyama et al. 1995) were all inactive to induce such a remarkable cell-dissociating effect (Shibata et al. 1998). At MBT, the cell dissociation was first observed at the region where SAMDC mRNA was microinjected (Fig. 1) and once it starts the whole embryos are soon dissolved completely. This embryo lysis was due to osmotic shock, since while embryos with dissociated cells kept in the hypotonic 0.1 X Steinberg’s solution autolyzed immediately, embryos with dissociated cells kept in the slightly hypertonic 1 X Steinberg’s solution did not dissolve, and cells which remained undissociated continued morphogenesis and formed structures like notochord and muscles (Shibata et al. 1998). The fact that extensive cell dissociation took place around the point of microinjection (Fig. 1) suggested non-uniform distribution of the injected SAMDC mRNA within the egg cytoplasm.

In SAMDC-overexpressed early blastulae, DNA, RNA and protein syntheses were all inhibited, but the most strongly inhibited was protein synthesis (Shibata et al. 1998). HPLC analysis revealed that SAM, a natural substrate of SAMDC, was greatly reduced in SAMDC mRNA-overexpressed embryos. When SAMDC mRNA was co-injected with either EGBG (ethylglyoxalbis(guanylhydrazone)), a specific inhibitor of SAMDC, or SAM, the substrate of SAMDC, cell dissociation was completely suppressed and embryos became normal swimming tadpoles (Shibata et al. 1998). We, therefore, concluded that overexpression of SAMDC results in SAM-deficiency, and this in turn results in severe inhibition of protein synthesis, which is most probably the direct cause for cell dissociation.

A remarkable aspect of these results was that cleavage proceeded normally and SAMDC- overexpressed embryos became normal-looking early blastulae at the correct timing, yet massive cell dissociation took place constantly at MBT, irrespective of the dosage of the injected mRNA (Shibata et al. 1998). Thus, as the amount of the injected mRNA decreased from 10,000 pg/egg to 100 pg/egg, the percentage of dissociated embryos decreased, yet those which were destined to undergo cell dissociation were all dissociated constantly at the time when control uninjected embryos reached MBT. This punctual cell dissociation at MBT suggested the occurrence of a clock mechanism which determines the timing of switching-on of this drastic effect.

## The Cell Dissociation Observed was Due to Execution of Apoptosis

**Figure 2:**
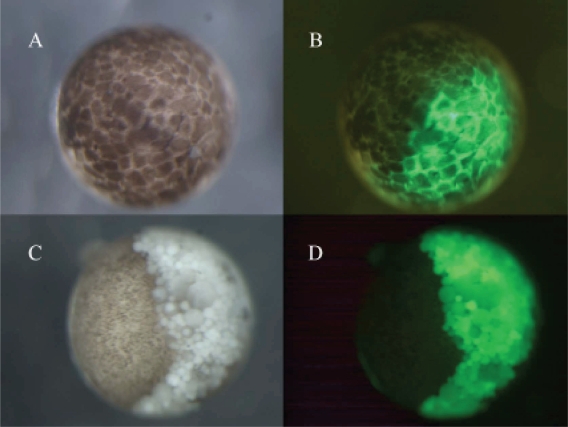
Apoptosis induced in a half portion of an embryo. Only one blastomere of a 2-celled embryo was injected with a mixture of SAMDC mRNA (1 ng/egg) and GFP mRNA (100 pg/egg), and embryos were filmed at early blastula (A, B) and early gastrula (C, D) stage using the visible light (A, C) and UV light (B, D). In B, cells expressing GFP, hence containing SAMDC mRNA, cleaved normally, but after MBT one half portion of the embryo which expressed GFP (D) underwent cell dissociation (C). (Kuroyanagi S, Shiokawa K, unpublished).

When we examined sectioned materials of SAMDC mRNA-injected blastulae at the beginning of dissociation, we detected a large number of dissociated cells not only in the perivitelline space but also within the blastocoel (Shibata et al. 1998). When we co-injected GFP (green fluorescent protein) and SAMDC mRNAs into only one blastomere of 2-celled embryos, only a half portion of embryos was dissociated at the late blastula stage (Fig. 2). It was apparent that all the dissociated cells were those which expressed GFP (stained green), indicating that only the cells that received SAMDC mRNA were dissociated. Electron microscopic analyses revealed that nuclei of such dissociated cells were fragmented into two or three portions (Kai et al. 2000), and in such SAMDC mRNA-injected embryos, a large number of cells became TUNEL-positive, and furthermore, DNA extracted from such embryos formed “ladders” on agarose gels (Kai et al. 2000). We injected SAMDC mRNA into uncleaved fertilized eggs and at the 2-cell stage further co-injected into one of the blastomeres GFP mRNA and mRNA of *Xenopus* Bcl-2 (Cruz-Reyes and Tata, 1995), an anti-apoptotic factor that suppressed apoptosis by inhibiting the release of cytochrome c from mitochondria (Yang et al. 1997; Kluck et al. 1997; Johnston et al. 2005). We found here that cell dissociation at MBT was suppressed only in the GFP-positive and hence Bcl-2-expressing half of the embryo (Kai et al. 2000). We, therefore, concluded that the cell dissociation observed was due to the execution of apoptosis. Thus, in this *Xenopus* embryonic system, SAMDC-overexpressed cells underwent apoptosis, rather than arresting cell cycles. This is interesting, since adult-type cells that happened to have DNA damages by γ-ray irradiation usually arrest cell cycles rather than undergoing apoptosis (Wroble and Sible, 2005).

## Apoptotic Cells are Observed also in Normally Developing Embryos

In normally-developing *Xenopus* embryos at MBT and after MBT, similar apoptotic cells have been reported to occur (Hensey and Gautier, 1998). In this case, however, the site of the appearance of apoptotic cells as well as the number of such cells differs from embryo to embryo even within the same batch (Hensey and Gautier, 1998), suggesting that such apoptotic cells are formed accidentally. When naturally-ovulated *Xenopus* fertilized eggs are cultured and handled in the laboratory, especially after removal of the jelly coat, embryos sometimes would receive mechanical shock during handling, and cells which received a strong shock might stop cell division and execute apoptosis. In such cases, apoptosis should appear as if it was executed spontaneously as described by Hensey and Gautier (1998).

## The Pre-Apoptotic Device Seems to Have Been Pre-Set Within the Egg

As with the origin of the pre-apoptotic device, there is an interesting experiment by Newmeyer et al. (1998), which reproduced apoptotic reaction *in vitro* in the extract of unfertilized eggs. These authors showed that nuclear events typical of apoptosis can be reproduced *in vitro* by incubating endogenous and exogenously-added nuclei in the cell-free extract *of Xenopus* eggs (Newmeyer et al. 1998). We, therefore, assume that there is a maternally-preset device of apoptosis program in *Xenopus* unfertilized eggs, though it may not be complete.

## Various Toxic Treatments Other Than SAMDC Overexpression Induce Similar Apoptotic Reactions

Experiments independently performed in other laboratories at about the same time have shown that quite similar cell dissociation due to apoptosis takes place in *Xenopus* blastulae when early cleavage stage embryos were treated with various toxic agents, such as γ-ray (Anderson et al. 1997; Hensey and Gautier, 1997), hydroxyurea (Stack and Newport, 1997), cycloheximide (Stack and Newport, 1997; Hensey and Gautier, 1997), and α-amanitin (Sible et al. 1997; Hensey and Gautier, 1997). Cells dissociated by these treatments were TUNEL-positive, and formed fragmented nuclei that contained fragmented DNA. Furthermore, onset of the cell dissociation executed by γ-ray exposure has been shown to be suppressed “partially” (the onset of the cell dissociation was postponed only by 2–3 hrs) by prior microinjection of Bcl-2 mRNA (Anderson et al. 1997; Hensey and Gautier, 1997). In more recent studies on the apoptosis induced by DNA-damaging agent like γ-ray, cyclin-dependent protein kinases have been shown to play important roles to switch on the apoptosis (Finkielstein et al. 2002; Carter and Sible, 2003; Carter et al. 2006; Wroble and Sible, 2005).

In our laboratory, we tested effects on *Xenopus* early embryos of several factors, and found that microinjection of p53 mRNA (Shiokawa et al. 2005), 5-aza-2′-deoxycytidine (5-Aza-CdR) (Kaito et al. 2001), and 5-methyl-2′-deoxycytidine-5′-triphosphate (5-methyl-dCTP) (Kaito et al. 2001) all induced execution of similar apoptosis. We also found that treatments of cleavage stage embryos with polyamines, colchicine, bufalin, brefeldin A, and moderately-high temperatures all induce cell dissociation which is similar to that induced by SAMDC as in the following:

### p53 mRNA

A tumor suppressor protein p53 is activated as a response to cellular lesions such as γ-ray-induced DNA damage, and activated p53 induces apoptotic factors such as Bax whose function is suppressed by Bcl-2 (Yang et al. 1997; Kluck et al. 1997). However, p53 is normally expressed in *Xenopus* early embryos and is in fact required for their normal development (Wallingford et al. 1997; Tchang et al. 1993), yet its overexpression in early embryos induces cell dissociation and embryo death at MBT (Hoever et al. 1994). We injected different amounts (10, 100, and 1000 pg/egg) of p53 mRNA into *Xenopus* fertilized eggs, and confirmed the findings by Hoever et al. (1994). Furthermore we found that the p53-induced cell dissociation was abolished by injection of mRNA for xdm-2 (mouse double minute-2) (Shiokawa et al. 1005), a negative regulator of p53 that directly binds to p53 (Momand et al. 1992). We also confirmed that DNA extracted from p53 mRNA-injected embryos formed ladders, and also that the onset of the cell dissociation was postponed for about 3 hrs by Bcl-2, although all embryos died before the late gastrula stage. Therefore, we concluded that p53, also, induces execution of the maternal program of apoptosis in *Xenopus* blastulae (Shiokawa et al. 2005).

### 5-Aza-CdR and 5-methyl-dCTP

**Figure 3:**
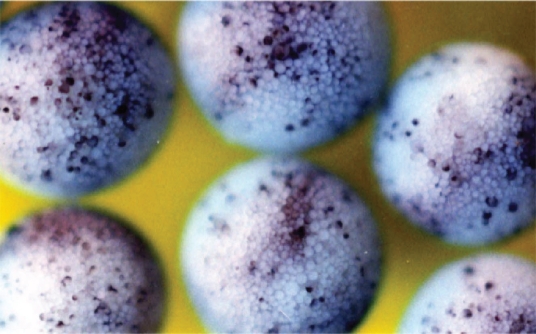
Cell dissociation induced by microinjection of 5-aza-2′-deoxycytidine (4 pmole/egg). Embryos were cultured in 1 X Steinberg’s solution and filmed at late blastula stage. From Kaito et al. (2001).

In embryos developing beyond MBT, failure of maintenance of methyltransferase leads to hypomethylation of DNA, and this in turn induces apoptotic cell death through activation of p53 pathway (Stancheva et al. 2001). 5-Aza-CdR induces hypomethylation of DNA (Jones, 1985). When this was injected into fertilized eggs (4 pmoles/egg), cleavage became slightly slower (at least by one cell cycle), but embryos continued to cleave until the midblastula stage, and then they were completely dissociated into cells and died (Fig. 3) (Kaito et al. 2001). This complete dissociation (Fig. 3) is different from that induced by SAMDC (Fig. 1), in the sense that the whole embryo was dissociated completely by 5-Aza-CdR (Fig. 3). We, therefore, assumed that, unlike SAMDC mRNA, 5-Aza-CdR spread easily and evenly throughout the egg cytoplasm. In this case, too, the dissociated cells had fragmented and TUNEL-positive nuclei with condensed chromatin, and ladder-forming DNA (Kaito et al. 2001). Furthermore, co-injection of 5-Aza-CdR and Bcl-2 mRNA postponed the onset of cell dissociation again for about 3 hrs; however the embryos eventually died at the gastrula stage. Also, when 5-Aza-CdR (0.4 pmoles) was co-injected with 10-fold larger amount of cytidine (CdR) (4 pmoles), a normal metabolite, the onset of the cell dissociation was postponed by ca. 3 hrs, and the embryos did not develop further. Using a specific antibody, we measured the level of 5-methyl-cytosine in the DNA from 5-Aza-CdR-injected blastulae, and found that DNA methylation was much reduced (Kaito et al. 2001). At early blastula stage, DNA, RNA, and protein syntheses were all inhibited (respectively, by ca. 70%, 40% or 30%) in 5-Aza-CdR-injected embryos. These results showed that 5-Aza-CdR induced hypomethylation of DNA and inhibition of macromolecular syntheses, especially the synthesis of DNA (Kaito et al. 2001).

5-Methyl-dCTP, on the other hand, induces hypermethylation of DNA (Holliday and Ho, 1991). When this was microinjected into *Xenopus* fertilized eggs (40 pmoles/egg), again cell dissociation and embryo death took place at MBT (Kaito et al. 2001). In this case, too, dissociated cells had fragmented and TUNEL-positive nuclei with condensed chromatin, and their DNA formed ladders, and co-injection of Bcl-2 mRNA postponed the onset of cell dissociation by about 3 hrs (Kaito et al. 2001). The co-injection of a 10-fold larger amount of a normal metabolite, dCTP (400 pmoles), also induced similar delay (2–3 hrs) of the onset of cell dissociation. In 5-methyl-dCTP-injected early blastulae the level of 5-methyl-cytosine in DNA was greatly increased (Kaito et al. 2001), and DNA and RNA synthesis was inhibited by ca. 40% and 20%, respectively, but protein synthesis was stimulated by ca. 30% (Kaito et al. 2001). These results suggests that hypermethylation of DNA induces apoptosis by suppressing gene expression.

### Polyamines, colchicines, bufalin, brefeldin A, and high temperature

We cultured *Xenopus* fertilized eggs and embryos in the medium which contained either putrescine, spermidine or spermine at 1–10 nmoles/ml, with a finding that these treatments also induce cell dissociation in embryos at MBT (Takubo S, Kondo T, Shiokawa K, unpublished results). The appearance of dissociated cells was quite similar to that of cells dissociated by overexpression of SAMDC.

**Figure 4:**
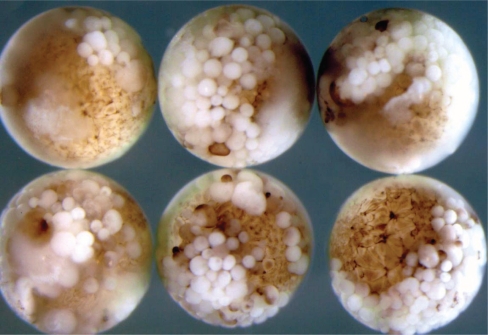
Apoptosis-like reaction in colchicine-treated *Xenopus* embryos. *Xenopus* fertilized eggs were cultured in 1 X Steinberg’s solution containing 1 mM colchicine. Embryos were filmed at early blastula stage. (Suwa M, Shiokawa K, unpublished).

When we cultured *Xenopus* fertilized eggs in the medium that contained colchicine (1 mM), we observed cell dissociation at early blastula stage (Suwa M, Shiokawa K, unpublished results) (Fig. 4). Here, the stage of the cell dissociation is before MBT, and the effect of colchicine may be due to more direct effect on the mitotic apparatus (Earnshaw and Cooke, 1991).

Bufalin, an inhibitor of Na^+^,K^+^-ATPase, is known to be an anticancer drug, and in a human leukemia cell line it induces apoptosis (Shiratsuki and Nakanishi, 1999). When we cultured 2-celled *Xenopus* embryos in the medium containing bufalin (100 μM), cell dissociation took place at early gastrula stage (Uchiyama H, Ikegami T, Shiokawa K unpublished results). The appearance of the dissociated embryos was similar to that induced by microinjection of 5-Aza-CdR.

Brefeldin A, a specific inhibitor of Golgi apparatus (Misumi et al. 1986), induces not only cell death by causing G_1_ arrest but also apoptosis through p53-independent pathway (Chapman et al. 1999). When we cultured 2-celled *Xenopus* embryos in the medium containing brefeldin A (40 μM), cell dissociation took place, again at early gastrula stage (Uchiyama H, Izumi Y, Misumi Y, and Shiokawa K unpublished results). This suggests that brefeldin A induces the execution of the maternal program of apoptosis just like bufalin.

When *Xenopus* embryos were cultured at moderately high temperature, development proceeds rapidly, however the tadpoles obtained are not abnormal. Thus, according to the Dettlaff’s rule (Dettlaff, 1964), if the length of time between the first cleavage to the second cleavage was expressed as tau (τ) and the stage of embryos cultured at different temperature was plotted as a function of τ, the time course of development follows exactly the same curve, irrespective of the difference in the temperature. However, this rule is not valid when temperature was higher than 32 °C. When *Xenopus* fertilized eggs were cultured at 34 °C, for instance, most of the embryos were dissociated into cells at early blastula stage, and died (Aso M, Mitsui K and Shiokawa K unpublished results). It appears that at moderately high temperature the maternal program of apoptosis is triggered and is executed.

## A Unique Aspect of SAMDC-Induced Apoptosis: Complete Rescue by Bcl-2

Bcl-2 inhibits the execution of apoptosis induced by γ-ray, α-amanitin and cycloheximide for 2–3 hrs, and thus “partially rescues” *Xenopus* embryos (Sible et al. 1997; Hensey and Gautier, 1997). When we tested the effect of Bcl-2 on the execution of apoptosis induced by 5-Aza-CdR, 5-methyl-dCTP, or p53 overexpression, the abrogation of apoptosis at MBT lasted only for 2–3 hrs, and embryos were eventually dissociated into cells and died at early gastrula stage (Kaito et al. 2001). Execution of apoptosis by 5-Aza-CdR or 5-methyl-dCTP was also suppressed for only 2–3 hrs when embryos were co-injected with normal metabolites, CR (cytidine) or dCTP, respectively (Kaito et al. 2001).

**Figure 5:**
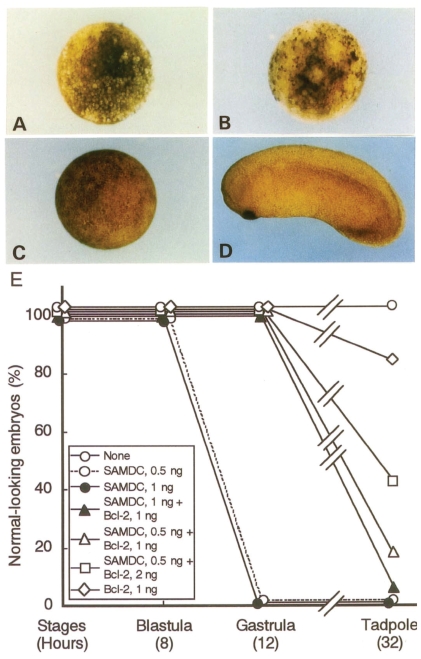
Rescue of SAMDC mRNA-injected *Xenopus* embryos by Bcl-2. When *Xenopus* fertilized eggs were injected with SAMDC mRNA (1 ng/egg) alone, embryos were dissociated shortly after MBT (A), and remained dissociated in 1 X Steinberg’s solution (B) even after 12 hrs (at which time control embryos reached stage 22 tailbud embryos). When fertilized eggs were co-injected with SAMDC mRNA (1 ng/egg) and Bcl-2 mRNA (2 ng/egg), cell dissociation did not take place at late blastula stage (C), and embryos developed to the tailbud stage (stage 22) (D). The extent of the rescue by Bcl-2 from SAMDC-induced apoptosis varied depending on the dosage-combination of SAMDC and Bcl-2 mRNAs (E). From Kai et al. (2000).

By a sharp contrast, apoptosis induced by SAMDC overexpression was completely suppressed by co-injection of Bcl-2 mRNA and rescued embryos continued to develop to the tailbud stage as in Figure 5 (Kai et al. 2000). In this experiment, it was confirmed by measuring the total SAMDC activity within the embryo, that the rescue was not due to inhibition of translation of the injected SAMDC mRNA (Kaito et al. 2001). The SAMDC-induced cell dissociation was also completely suppressed by co-injection of SAM (normal substrate of SAMDC) (Shibata et al. 1998), or EGBG (a specific inhibitor of SAMDC) (Shibata et al. 1998). Thus, the effect of SAMDC, a naturally occurring enzyme, is quite unique, in the sense that its apoptosis-inducing effect can be completely suppressed.

We followed the level of the microinjected SAMDC mRNA (1 ng/egg) within the embryo by northern blot analysis, and found that its level decreases rapidly to ca. one-third of its initial level by late neurula stage. We also found that the level of the overexpressed SAMDC decreased rapidly by the neurula stage to ca. one-third of its level at the MBT, a finding which is consistent with the high turnover nature of SAMDC (Heby and Persson, 1990). The rapid decrease of both SAMDC and its mRNA during gastrula through neurula stages probably explains why SAMDC mRNA-induced apoptosis can be completely suppressed (Kai et al. 2000).

## Involvement of Caspases in SAMDC-Induced Apoptosis

Enzymes involved in the execution of apoptosis are caspases which constitute a cysteine protease enzyme family (Thornberry, 1997; Salvesen and Dixit, 1997). Previous studies showed that cell lysates of γ-ray-irradiated or hydroxyurea-treated *Xenopus* embryos have activity to cleave poly-ADP-ribose polymerase (PARP) (Hensey and Gautier, 1997; Stack and Newport, 1997), a substrate of most caspases, including caspases-3 and -7 (Thornberry, 1997). Also, a synthetic peptide inhibitor which inhibits caspase-9 and caspase-3 has been reported to postpone the onset of apoptosis in cycloheximide-treated and γ-ray-irradiated *Xenopus* embryos (Hensey and Gautier, 1997; Stack and Newport, 1997). It is known that caspase-1 and possibly caspases-4 and -5 are primarily involved in procytokine activation, whereas other caspases promote pathways to apoptosis (Salvesen and Dixit, 1997). However, little has been known about caspases involved in the execution of apoptosis in *Xenopus* early embryos. We therefore examined caspases-9, -8 and -3 and their mRNA in *Xenopus* early embryos as in the following:

### Caspase-9

When *Xenopus* fertilized eggs were injected with SAMDC mRNA together with a synthetic peptide inhibitor specific for caspase-9 (Ac-LEHD-CHO) or caspase-1 (Ac-YVAD-CHO), SAMDC mRNA-induced apoptosis was suppressed dosage-dependently by the inhibitor for caspase-9, but not by the inhibitor for caspase-1. Embryos rescued here developed up to normal tadpoles. Since caspase-9 without its active site competes with wild-type caspase-9 at the step of binding to Apaf-1 and works as a dominant-negative type mutant (Li et al. 1997), the cysteine residue at the active site in caspases-9 and -1 (Nakajima et al. 2000; Yaoita and Nakajima, 1997) was replaced with phenylalanine (Takayama et al. 2004). When these mutated mRNAs were injected into fertilized eggs together with SAMDC mRNA, the mutated mRNA of caspase-9, but not caspase-1, completely suppressed SAMDC-induced apoptosis (Takayama et al. 2004). We then prepared lysates from SAMDC mRNA-injected embryos at cleavage (still normally cleaving) and late blastula (already in the apoptotic phase) stages, and incubated them with *in vitro* translated ^35^S-labeled procaspases. We found here that the lysate from the late blastulae, but not from cleavage stage embryos, contained the activity to cleave ^35^S-labeled procaspase-9 (Takayama et al. 2004). This suggested that the activity to process procaspase-9 appears at the blastula stage in SAMDC-overexpressed embryos. When we microinjected large amounts (100–1000 pg/egg) of caspase-9 mRNA alone into *Xenopus* fertilized eggs, apoptosis was executed dosage-dependently. The execution of this apoptosis was delayed by only 2–3 hrs by Bcl-2 and embryos died at the gastrula stage (Takayama et al. 2004).

### Caspase-8

In the mammalian apoptotic system, caspase-8 is activated by apoptotic receptors such as FAS and TNF-receptors, and this activates another apoptotic factor Bid, whose function can be canceled by Bcl-XL and Bcl-2 (Yang et al. 1997; Kluck et al. 1997; Johnston et al. 2005). When we injected SAMDC mRNA together with a synthetic peptide-inhibitor for caspase-8 (Ac-IETD-CHO) into *Xenopus* fertilized eggs, the inhibitor suppressed apoptosis dosage-dependently (Shiokawa et al. 2005). When we made a dominant-negative type mutant of caspase-8 by replacing the cysteine in the active site with phenylalanine, and injected it together with the wild-type SAMDC mRNA into fertilized eggs, execution of SAMDC-induced apoptosis was again completely suppressed. These results strongly suggest that SAMDC induces apoptosis *via* a step which involves activation of caspase-8 (Shiokawa et al. 2005).

We prepared lysates of SAMDC-overexpressed embryos at cleavage and blastula stage, and incubated them with *in vitro* synthesized ^35^S-labelled procaspase-8. We found here that the lysate from SAMDC mRNA-injected embryos at the blastula stage, but not at the cleavage stage, cleaved procaspase-8 (Shiokawa et al. 2005). This result suggests that procaspase-8 is converted into active caspase-8 in SAMDC-overexpressed embryos at the blastula stage. We assume that the activation of caspase-8 occurs prior to the activation of caspase-9 as in other systems (Slee et al. 1999). In this experiment, too, we found that microinjection of a relatively high dose of caspase-8 into *Xenopus* fertilized eggs induced cell dissociation and embryo death at MBT. Since DNA from caspase-8-overexpressed embryos formed ladders, and the onset of cell dissociation was delayed by about 3 hrs by co-injection of Bcl-2 mRNA, we concluded that the cell dissociation induced by microinjection of caspase-8 was also due to apoptosis (Shiokawa et al. 2005).

### Caspase-3

When we injected SAMDC mRNA (0.5 ng/egg) together with a specific peptide inhibitor for caspase 3 (Z-D(OMe)GMD(OMe)FMK) (0.5 ng/egg) into *Xenopus* fertilized eggs, ca. 70% of the co-injected embryos were rescued to develop beyond neurula stage (Kuroyanagi S, Shiokawa K, unpublished). We, therefore, assumed that caspase-3 is also involved as an executive caspase in the SAMDC-induced apoptosis, probably at a step downstream of caspase-9 as in other systems (Slee et al. 1999).

## Comparison of Caspase Systems in SAMDC-Induced and p53-Induced Apoptotic Embryos

When we co-injected into *Xenopus* fertilized eggs the caspase-9-specific peptide inhibitor (Ac-LEHD-CHO) and p53 mRNA, apoptosis did not take place at MBT. When we co-injected mRNAs for the dominant-negative type mutant of caspase-9 and p53, apoptosis did not take place at MBT. In both cases, however, the suppression of apoptosis was only 2–3 hrs, and embryos died at the early gastrula stage. When we co-injected p53 mRNA and the caspase-8-specific peptide inhibitor, the inhibitor did not suppress p53-induced apoptosis. Also when we injected p53 mRNA together with the dominant-negative type mutant of caspase-8, the p53-induced apoptosis was not suppressed (Shiokawa et al. 2005). Furthermore, when we incubated ^35^S-labeled procaspase-9 with the lysate of p53 mRNA-overexpressed embryos at cleavage (normally dividing) or blastula (already apoptotic) stage, the lysate from blastulae, but not that from cleavage stage embryos, cleaved the procaspase-9. On the other hand, the lysate of p53-induced apoptotic blastulae did not cleave ^35^S-labeled procas-pase-8, although the lysate of SAMDC-induced apoptotic blastulae cleaved ^35^S-labeled procaspase-8 (Shiokawa et al. 2005).

These results indicate that in SAMDC mRNA-induced apoptotic embryos activities to cleave both procaspase-8 and -9 appear before the execution of apoptosis, whereas in p53 mRNA-induced apoptotic embryos the activity to process caspase-9, but not caspase-8, appears. We, therefore, conclude that SAMDC-induced apoptosis is executed through the steps involving both caspase-8 and caspase-9; whereas p53 induced apoptosis is executed *via* activation of caspase-9 without the step that involves the activation of caspase-8.

## Developmental Changes in the Level of Caspase mRNAs

**Figure 6:**
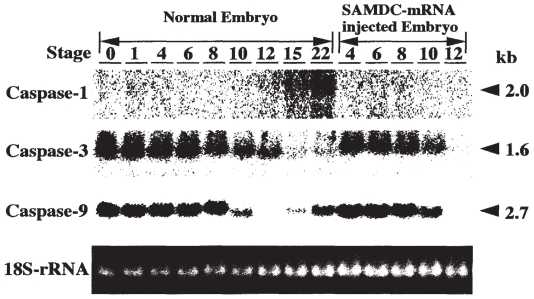
Northern blot analysis of caspase mRNAs in *Xenopus* embryos. Fertilized eggs were injected with either SAMDC mRNA (100 pg/egg) or distilled water, and RNAs were isolated from embryos, and subjected to northern blot analysis. From Takayama et al. (2004).

We analyzed RNAs extracted from SAMDC mRNA-injected and p53 mRNA-injected embryos by northern blot analysis. In uninjected embryos, we detected ca. 2.7 Kb, 2.0 Kb, and 1.6 Kb signals as caspase-9, caspase-1, and caspase-3 mRNA, respectively (Fig. 6) (Takayama et al. 2004). Caspase-9 mRNA occurred most abundantly in unfertilized eggs, and the level was maintained until MBT, once decreased at gastrula stage, was then increased from the late neurula stage. By contrast, caspase-1 mRNA was detected first at the late gastrula stage, and its level increased thereafter, indicating that caspase-1 mRNA is expressed only in post-gastrular embryos. This result may be correlated to the fact that caspase-1 is reportedly involved in neuronal cell death (Friedlander et al. 1997). Caspase-3 mRNA occurred as a maternal mRNA throughout cleavage stage, but its level was lower than that of caspase-9. The expression of these three caspase mRNAs was not appreciably affected by injection of SAMDC mRNA (Fig. 6) throughout early stages.

**Figure 7:**
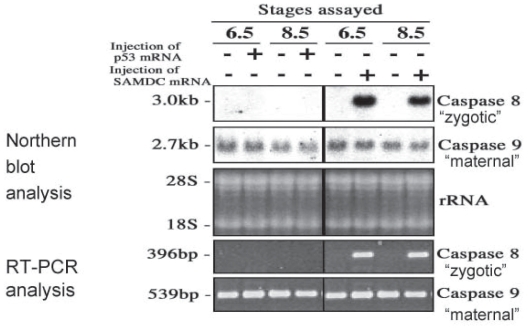
Northern blot and RT-PCR analyses for caspase-8 and -9 mRNAs in p53 mRNA- or SAMDC mRNA-injected embryos. Fertilized eggs were injected (+) or not (−) injected with p53 or SAMDC mRNA (1 ng each/embryo), and cultured in 1 X Steinberg’s solution. RNAs were isolated from embryos at stage 6.5 (late cleavage stage) or stage 8.5 (midblastula stage). Upper white panels: RNAs separated on a 1% agarose gel containing formaldehyde were transferred to a nylon membrane, and hybridized with ^32^P-labelled specific probes for *Xenopus* caspase-8 and 9. Middle black panel: 28S and 18S rRNAs stained with ethidium bromide on 1% agarose gel. This profile is before blotting. Lower black panels: RNAs were subjected to RT-PCR. The signal obtained for caspase-8 and caspase-9 mRNA was 396 bp and 539bp, respectively. Caspase-8 mRNA expression was induced only in SAMDC mRNA-injected cleavage stage and midblastula stage embryos, whereas caspase-9 mRNA occurred as a maternally-provided RNA. From Shiokawa et al. (2005).

**Figure 8:**
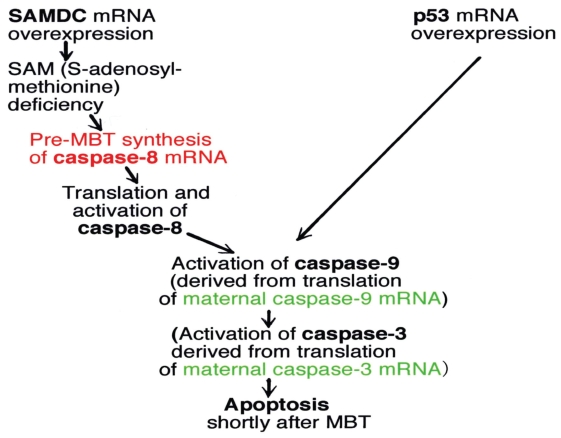
A model which shows sequence of events in activation of caspase system in SAMDC mRNA-overexpressed and p53 mRNA-overexpressed embryos, with special reference to the recruitment of mRNAs.

Caspase-8 mRNA (3.0 Kb) was not detected from cleavage through blastula stages in both uninjected embryos and in p53-induced apoptotic embryos. However, this mRNA was newly expressed in SAMDC mRNA-injected embryos both at cleavage (non-apoptotic) and late blastula (already apoptotic) stages (Fig. 7). We performed RT-PCR analysis, and confirmed the result of the northern blot analysis (Fig. 7). Thus, caspase-8 mRNA is newly synthesized during cleavage stage (pre-MBT stage) when embryos were overexpressed with SAMDC mRNA, but not p53 mRNA (Shiokawa et al. 2005). The sequence of events in activation of caspases and supply of their mRNAs in early stage *Xenopus* embryos can be summarized as in Fig. 8.

## When the Number of Damaged Cells is Not Large, They are Segregated into the Blastocoel and Undergo Apoptosis within the Blastocoel, but Embryos Do Not Stop Development

When SAMDC mRNA was injected into a blas-tomere of embryos at 2- to 4-cell stages, embryos died due to massive cell dissociation shortly after MBT (Shiokawa et al. 1998; Kai et al. 2000). However, when SAMDC mRNA was injected into only one blastomere in embryos at 8- to 32-cell stages, almost all the injected embryos became tadpoles, without showing any sign of apoptosis at MBT (Kai et al. 2003). At the beginning, this result was embarrassing to us, because it appeared that apoptosis had not been executed in those cells which were derived from the SAMDC mRNA-injected blastomere. We then co-injected mRNAs for SAMDC and GFP into one of the blastomeres in embryos at 8–32 cell stage and followed the fate of the descendant cells using GFP as a lineage tracer (Kai et al. 2003).

**Figure 9:**
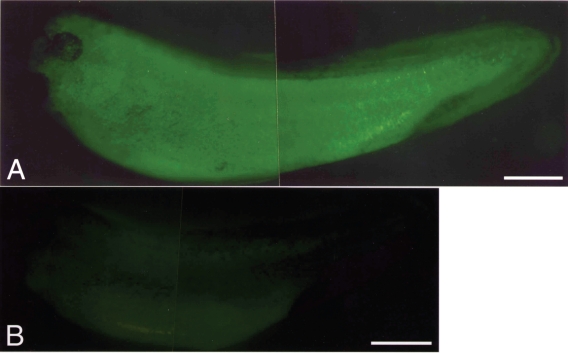
GFP-tracing of SAMDC mRNA-injected cells at the tadpole stage. Embryos were injected with processing-defective (A) or wild-type (B) SAMDC mRNA together with GFP mRNA into one animal side blastomere at the 8-cell stage, and GFP luminescence was examined at the tadpole stage (32 hrs post-fertilization). Note that the embryo in B is shorter in body length than that in A due to apoptotic loss of a certain amount of cell mass at MBT within the blastocel. Both embryos were too large to be taken in a photograph, so embryos were taken in two photographs and tadpoles were constructed by combining the two photographs together. The bar is 5 mm. From Kai et al. (2003).

In this experiment, we injected into the control embryos, a processing-defective *Xenopus* SAMDC mRNA, which neither increased SAMDC activity within the embryo nor induced apoptosis (Kai et al. 2003). From cleavage to early blastula stages we found no difference in the outer appearance of embryos and in the approximate size of GFP-expressing cell mass between mutated mRNA-injected and wild-type mRNA-injected embryos. At late blastula stage, however, while widely-spread luminescent cells were seen from outside in the mutated mRNA-injected embryos, no such luminescent cells were seen on the surface of the wild-type mRNA-injected embryos (Kai et al. 2003). Here, we disrupted embryos and examined the inside of the embryos, and we found many dissociated luminescent cells within the blastocoel in the wild-type mRNA-injected embryos, but not in the mutated mRNA-injected embryos. Nuclei of such dissociated cells were fragmented and their chromatin was condensed, indicating that these were apoptotic cells (Kai et al. 2003). At swimming tadpole stage, whole bodys of the mutant mRNA-injected embryos were green due to many luminescent cells throughout the embryo, but no luminescent cells were found in the wild-type mRNA-injected embryos (Fig. 9). In this experiment, however, we noticed that tadpoles derived from wild-type mRNA-injected embryos were shorter in body length as compared with the controls (Fig. 9). In other cases, tadpoles obtained from wild-type mRNA-injected embryos often had small head (sometimes acephaly), small trunk and tail, and body axis bending. Thus, when a relatively small number of cells are damaged in cleavage stage embryos of *Xenopus laevis*, the damaged cells are segregated into the blastocoel at MBT and undergo apoptosis in the blastocoel, and embryos continue development, although the tadpoles obtained are not necessarily normal.

## Effects of SAMDC mRNA Injection on Morphogenesis

**Figure 10:**
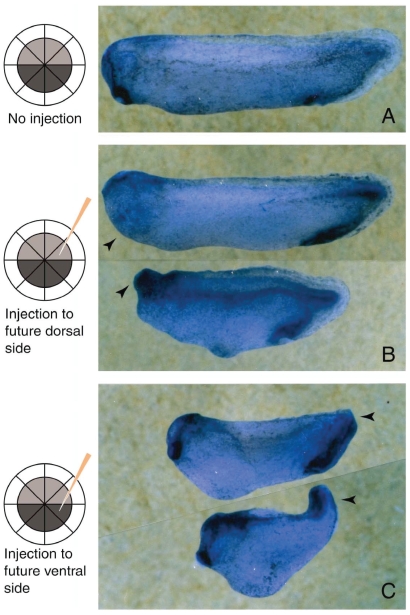
Effects on development of SAMDC mRNA injection into an animal side blastomere of either future dorsal or ventral side of the 16-cell stage embryo. Embryos were injected with SAMDC mRNA (130 pg) into one animal side blastomere of either future dorsal (B) or ventral (C) side at 16-cell stage as schematically shown in the diagram in the left, and cultured until the tailbud stage. (A) A control uninjected embryo. In B, the cement gland is missing (arrowheads), and furthermore, head part is absent (acephaly) (lower arrowhead). In C, posterior and ventral structures such as trunk and tail are poorly developed (arrowheads), or tail is curved upwards (lower arrowhead). From Kai et al. (2003).

Since presumptive fates of blastomeres are known from the pigmentation pattern at 2- to 16-cell stages (Shiokawa and Yamana, 1979; Moody, 1987), we injected wild-type SAMDC mRNA into a blastomere at the future dorsal side at 16-cell stage, and followed the development of the injected embryos. The result obtained showed that although all embryos became tadpoles, ca. 40% of them had strong defects in cement gland and head part. Conversely, when we injected wild-type SAMDC mRNA into a blastomere at the future ventral side at 16-cell stage, ca. 30% of embryos developed into embryos with poorly developed trunk and tail (Kai et al. 2003) (Fig. 10). These results show that elimination of cells in the specific embryonic regions at MBT is reflected in the specific defect in tadpoles.

## Maternal Program of Apoptosis as a “fail-safe” Mechanism of Early Embryonic Development

Eukaryotic cells have a suicide program, termed apoptosis, and this has a function to remove damaged cells, and is also essential for tissue homeostasis and morphogenesis. Thus, in *Xenopus laevis*, studies of apoptosis have been focused on the widespread cell death as in the process of tail regression during metamorphosis (Nishikawa et al. 1989; Tata et al. 1991; Wang and Brown, 1993; Shi et al. 1996). In this review we focused on data which show that such apoptotic system is also essential for *Xenopus* early embryogenesis, although this apoptosis seems to be a mechanism which has different physiological significance as compared with the apoptosis or the programmed cell death in the process of brain segmentation (Shinga et al. 2001) and tail regression (Nishikawa et al. 1989; Tata et al. 1991; Wang and Brown, 1993; Shi et al. 1996). In this connection it is interesting that Johnston et al. (2005) showed that by overexpressing Bcl-X_L_ in transgenic *Xenopus* embryos that apoptosis executed by γ-ray at MBT can be abrogated by Bcl-X_L_, whereas the transgene did not prevent the thyroid hormone-induced cell death during metamorphosis in *Xenopus* tadpoles. However, the apoptotic program in early cleavage stage embryos may be a universal phenomenon, since a similar apoptotic reaction occurs also in zebrafish embryos (Ikegami et al. 1999).

**Figure 11:**
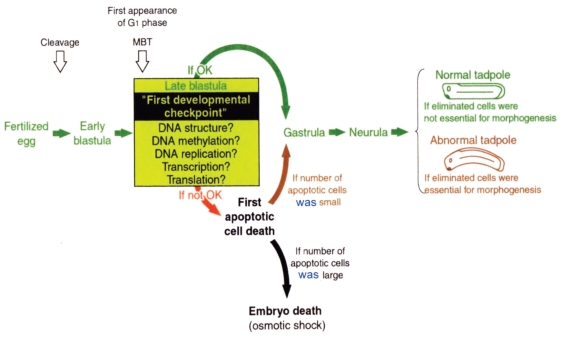
A model which shows how early development proceeds. This model suggests possible occurrence of apoptotic check point which functions as a surveillance or “fail-safe” mechanism in *Xenopus* early embryonic development. Fertilized eggs cleave rapidly until the early blastula stage. At MBT, the “first developmental checkpoint” comes when G1 phase first appears. We assume that this check mechanism determines cell-autonomously if the cell continues or stops development to be eliminated by execution of the maternally-inherited program of apoptosis. However, even when apoptosis was executed, embryos follow two different courses. If the number of apoptotic cells is large, the whole embryo stops development and dies. However, if the number of apoptotic cells is small, they are confined within the blastocoel and the embryo itself continues on development. Cells to be eliminated are segregated into the blastocoel. If such cells came out to the perivitteline space, the whole embryo will be dissolved due to osmotic shock (eggs are laid in the water), and the maternally-inherited program of apoptosis will not serve as a fail-safe mechanism.

The execution of apoptosis in very early development in *Xenopus* could be summarized as in Figure 11. As Hensey and Gautier (1998) pointed out, it appears that embryonic cells check themselves to see if they are capable of continuing further development at MBT, when G_1_ phase first appears in the cell cycle. If some cells find themselves physiologically aberrant, they disappear from the embryo by executing the apoptotic program. The cellular activities to be checked here seem to be diverse, including the level of SAM, DNA structure, DNA replication, DNA methylation, RNA transcription, and translation, as expected from the results obtained using SAMDC mRNA, γ-ray, hydroxyurea, α-amanitin, cycloheximide, and 5-Aza-CdR (Shibata et al. 1998; Anderson et al. 1997; Sible et al. 1997; Hensey and Gautier, 1997; Stack and Newport, 1997; Kaito et al. 2001). We assume that the maternal program of apoptosis in *Xenopus* eggs constitutes a surveillance or a “fail-safe” mechanism for normal development to check and eliminate damaged cells shortly after MBT to save the rest of the embryo (Shiokawa et al. 2000; Kai et al. 2003).

SAMDC overexpression depletes SAM from cells (Shibata et al. 1998), and this might cause the inhibition of mRNA cap methylation. In our very early experiment to label *Xenopus* embryonic cells at cleavage and blastula stages, very extensive activities were detected in mRNA cap methylation in spite of the fact that there was little 2′-O-methylation in the high-molecular-weight RNAs (Shiokawa et al. 1981a, b). In this connection, we recently performed experiments to co-inject SAMDC mRNA and cap analogue (m^7^GpppG) into *Xenopus* fertilized eggs, and found that ca. 50% of the co-injected embryos were rescued and developed into tadpoles (Higo T, Takayama E, Shiokawa K, unpublished results). This issue will need to be examined more extensively, but at this stage we tentatively interpreted these results as suggesting that overexpression of SAMDC suppresses mRNA cap methylation, and this suppression could be a reason for the inhibition of protein synthesis that leads to the execution of apoptosis.

## Three Gene Expression Patterns that Characterize Early *Xenopus* Embryogenesis

Finally, it is worth referring here to the appearance of caspase-8 mRNA in SAMDC mRNA-injected cleavage stage embryos. For many years, most *Xenopus* researchers believed that pre-MBT embryos are transcriptionally totally silent (Newport and Kirschner, 1982; Yasuda and Schbiger, 1992; Andeol, 1994). In this connection, it is true that rRNA synthesis is in fact totally absent during pre-MBT stage, but starts right after MBT as shown by ourselves in 1981 (one year before the presentation of the theory of the total absence of transcription in pre-MBT *Xenopus* embryos) (Shiokawa et al. 1981a, b). In 1987, however, we reported that during pre-MBT stage heterogeneous non-mitochondrial mRNA-like RNA is labeled with ^3^H-uridine (Nakakura et al. 1987), although it is not yet clear to what extent such heterogeneously-labeled RNA is processed to mature mRNA to be transported to the cytoplasm. Furthermore, in 2002 it was reported that nodal-related TGF-β superfamily member genes, Xnr5 and Xnr6, are transcribed during the pre-MBT stage, and suppression of this transcription interferes with post-gastrular morphogenetic movements (Yang et al. 2002). There is another striking change in the nuclear transcriptional activity before and after MBT. It is the onset of the burst of tRNA synthesis at MBT, which is estimated to be ca. 100 times faster than that in the postgastrula embryos. Thus, tRNA synthesis is activated greatly at MBT and its per-cell rate w as calculated on a per-cell basis (Shiokawa et al. 1981a, b).

**Figure 12:**
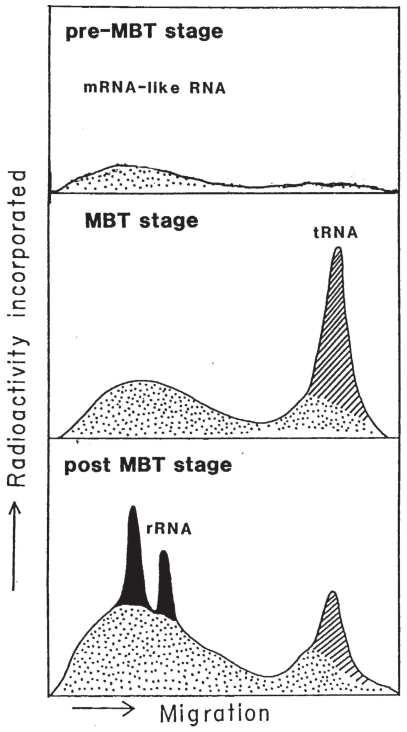
Three characteristic profiles of RNA synthetic patterns as studied by gel electrophoresis of radioactively-labeled RNA at each stage. Dotted, shaded and black areas are for the product of RNA polymerase II, III, and I, respectively, which are heterogeneous mRNA-like RNA, tRNA, and rRNA, respectively. Distance of migration and amount of radioactivity are in arbitrary units. From Shiokawa et al. (1941).

We proposed a model that characterizes RNA synthetic patterns in early *Xenopus* embryogenesis as in Figure 12. Preblastula stage is characterized by low level of the synthesis of heterogeneous high molecular weight RNA; and MBT is characterized by strong activation of tRNA and mRNA; and post-MBT stage is characterized by onset and increasing activity of rRNA synthesis (Fig. 12). Therefore, caspase-8 mRNA appears to be the third species of polymerase II-transcribed RNA that has been reported to be expressed in pre-MBT stage (Shiokawa et al. 2005).

## References

[b1-grsb-2008-213] Andeol YA 1994 Early transcription in different animal species: Implication for transition from maternal to zygotic control in development Roux’s Arch. Dev. Biol 204 3 10 10.1007/BF00189062 28305800

[b2-grsb-2008-213] Anderson JA Lewellyn AL Maller JL 1997 Ionizing radiation induces apoptosis and elevates cyclin A1-Cdk2 activity before but not after the midblastula transition in *Xenopus* Mol. Biol. Cell 8 1195 206 9243501 10.1091/mbc.8.7.1195 PMC276146

[b3-grsb-2008-213] Brown DD Littna E 1964 RNA synthesis during the development of *Xenopus* laevis, the African clawed toad J. Mol. Biol 8 669 87 14187393 10.1016/s0022-2836(64)80116-9

[b4-grsb-2008-213] Brown DD Littna E 1966 Synthesis and accumulation of DNMA-like RNA during embryogenesis of *Xenopus laevis* J. Mol. Biol 20 81 94 6008162 10.1016/0022-2836(66)90119-7

[b5-grsb-2008-213] Busby SJ Reeder RH 1983 Spacer sequences regulate transcription of ribosomal gene plasmids injected into *Xenopus* embryos Cell 34 989 96 6627394 10.1016/0092-8674(83)90556-1

[b6-grsb-2008-213] Carter AD Sible JC 2003 Loss of XChk1 function triggers apoptosis after the midblastula transition in *Xenopus* embryos Mech. Dev 120 315 23 12591601 10.1016/s0925-4773(02)00443-4

[b7-grsb-2008-213] Carter AD Wroble BN Sible JC 2006 Cyclin A1/Cdk2 is sufficient but not required for the induction of apoptosis in early *Xenopus* embryos Cell. Cycle 5 2230 6 16969089 10.4161/cc.5.19.3262

[b8-grsb-2008-213] Chapman JR Tazaki H Mallough C 1999 Mechanism of brefeldin A-induced growth inhibition and cell death in human prostatic carcinoma cells Mol. Urol 3 11 6 10851291

[b9-grsb-2008-213] Curz-Reyes J Tata JR 1995 Cloning, characterization and expression of two *Xenopus* bcl-2-like cell-survival genes Gene 158 171 9 7607538 10.1016/0378-1119(95)00159-4

[b10-grsb-2008-213] Davidson EH 1976 Gene Activity in Early Development New York Academic Press

[b11-grsb-2008-213] Davis RH Morris DR Coffino P 1992 Sequestered end products and enzyme regulation: the case of ornithine decarboxylase Microbiol. Rev 56 280 90 1620066 10.1128/mr.56.2.280-290.1992 PMC372868

[b12-grsb-2008-213] Dettlaff TA 1964 Cell division, duration of interkinetic states, and differentiation in early stages of embryonic development Adv. Morphog 3 323 62 14284233 10.1016/b978-1-4831-9950-4.50011-4

[b13-grsb-2008-213] Finkielstein CV Chen LG Maller JL 2002 A role for G1/S cyclin-dependent protein kinases in the apoptotic response to ionizing radiation J. Biol. Chem 277 38476 85 12176996 10.1074/jbc.M206184200

[b14-grsb-2008-213] Earnshaw WC Cooke CA 1991 Analysis of the distribution of the INCENPs throughout mitosis reveals the existence of a pathway of structural changes in the chromosomes during metaphase and early events in cleavage furrow formation J. Cell. Sci 98 443 61 1860899 10.1242/jcs.98.4.443

[b15-grsb-2008-213] Etkin LD Balcells S 1985 Transformed *Xenopus* embryos as a transient expression system to analyze gene expression at the midblastula transition Dev. Biol 108 173 8 3871711 10.1016/0012-1606(85)90019-3

[b16-grsb-2008-213] Friedlander RM Gagliardini V Hara H 1997 Expression of a dominant negative mutant of interleukin-1β converting enzyme in transgenic mice prevents neuronal cell death induced by trophic factor withdrawal and ischemic brain injury J. Exp. Med 185 933 40 9120399 10.1084/jem.185.5.933 PMC2196165

[b17-grsb-2008-213] Graham CF Morgan RW 1966 Changes in the cell cycle during early amphibian development Dev. Biol 14 439 60

[b18-grsb-2008-213] Guirard BM Snell EE 1964 Effect of polyamine structure on growth stimulation and spermine and spermidine content of lactic acid bacteria J. Bacteriol 88 72 80 14197908 10.1128/jb.88.1.72-80.1964 PMC277258

[b19-grsb-2008-213] Gurdon JB 1988 A community effect in animal development Nature 336 772 4 3205305 10.1038/336772a0

[b20-grsb-2008-213] Gurdon JB Brown DD 1965 Cytoplasmic regulation of RNA synthesis and nucleolar formation in developing embryos *Xenopus* laevis J. Mol. Biol 12 27 35 14343286 10.1016/s0022-2836(65)80279-0

[b21-grsb-2008-213] Heasman J 2006 Patterning the early *Xenopus* embryo Development 133 1205 17 16527985 10.1242/dev.02304

[b22-grsb-2008-213] Heby O Persson L 1990 Molecular genetics of polyamine synthesis in eukaryotic cells Trends Biochem. Sci 15 153 8 2187296 10.1016/0968-0004(90)90216-x

[b23-grsb-2008-213] Hensey C Gautier J 1997 A developmental timer that regulates apoptosis at the onset of gastrulation Mech. Dev 69 183 95 9486540 10.1016/s0925-4773(97)00191-3

[b24-grsb-2008-213] Hensey C Gautier J 1998 Programmed cell death during *Xenopus* development: A spatial-temporal analysis Dev. Biol 203 36 48 9806771 10.1006/dbio.1998.9028

[b25-grsb-2008-213] Hoever M Clement JH Wedlich D 1994 Overexpression of wild-type p53 interferes with normal development in *Xenopus* laevis embryos Oncogene 9 109 20 8302570

[b26-grsb-2008-213] Holloday R Ho T 1991 Gene silencing in mammalian cells by uptake of 5-methyl deoxycytidine-5′-triphosphate Somat Cell. Mol. Genet 17 537 42 1722591 10.1007/BF01233618

[b27-grsb-2008-213] Ikegami R Hunter P Yager TD 1999 Developmental activation of the capability to undergo checkpoint-induced apoptosis in the early zebrafish embryo Dev. Biol 209 409 33 10328930 10.1006/dbio.1999.9243

[b28-grsb-2008-213] Johnston J Chan R Calderon-Segura M 2005 The roles of Bcl-XL in modulating apoptosis during development of *Xenopus laevis* BMC Dev. Biol 5 1 16 16185362 10.1186/1471-213X-5-20 PMC1262703

[b29-grsb-2008-213] Jones PA 1985 Altering gene expression with 5-azacytidine Cell 40 485 6 2578884 10.1016/0092-8674(85)90192-8

[b30-grsb-2008-213] Kai M Higo T Yokoska J 2000 Overexpression of S-adenosylmethionine decarboxylase (SAMDC) activates the maternal program of apoptosis shortly after MBT in *Xenopus* embryos Int. J. Dev. Biol 44 507 10 11032186

[b31-grsb-2008-213] Kai M Kaito C Fukamachi H 2003 Overexpression of S-adenosylmethionine decarboxylase (SAMDC) in *Xenopus* embryos activates maternal program of apoptosis as a “fail-safe” mechanism of early embryogenesis Cell. Res 13 147 58 12862315 10.1038/sj.cr.7290159

[b32-grsb-2008-213] Kaito C Kai M Higo T 2001 Activation of the maternally preset program of apoptosis by microinjection of 5-aza-2′-deoxycytidine and 5-methyl-2′-deoxycytidine-5′-triphosphate in *Xenopus laevis* embryos Develop. Growth Differ 43 383 90 10.1046/j.1440-169x.2001.00579.x 11473545

[b33-grsb-2008-213] Kane DA Kimmel CB 1993 The zebrafish midblastula transition Development 119 447 56 8287796 10.1242/dev.119.2.447

[b34-grsb-2008-213] Kluck RM Bossy-Wetzel E Green DR 1997 The release of cytochrome c from mitochondria: a primary site for Bcl-2 regulation of apoptosis Science 275 1132 6 9027315 10.1126/science.275.5303.1132

[b35-grsb-2008-213] Kondo M Tashiro K Fujii G 1991 Actibin receptor mRNA is expressed early in *Xenopus* embryogenesis and the level of the expression affects in the body axis formation Biochem. Biophys. Res. Commun 181 684 90 1661587 10.1016/0006-291x(91)91245-8

[b36-grsb-2008-213] Li P Nijhawan D Budihardjo I 1997 Cytochrome C and dATP-dependent formation of Apaf-1/caspase-9 complex initiates an apoptotic cascade Cell 91 479 89 9390557 10.1016/s0092-8674(00)80434-1

[b37-grsb-2008-213] Minoura I Nakamura H Tashiro K 1995 Stimulation of circus movement by activin, bFGF and TGF-beta 2 in isolated animal cap cells of *Xenopus* laevis Mech. Dev 49 65 9 7748790 10.1016/0925-4773(94)00303-5

[b38-grsb-2008-213] Misumi Y Misumi Y Miki K 1986 Novel blockade by brefeldin A of intracellular transport of secretary proteins in cultured rat hepatocytes J. Biol. Chem 261 11398 403 2426273

[b39-grsb-2008-213] Momand J Zambetti GP Olson DC 1992 The mdm-2 oncogene product forms a complex with the p53 protein and inhibits p53-mediated transactivation Cell 69 1237 45 1535557 10.1016/0092-8674(92)90644-r

[b40-grsb-2008-213] Moody SA 1987 Fates of the Blastomeres of the 16-Cell. Stage *Xenopus* Embryo Dev. Biol 119 560 78 3803718 10.1016/0012-1606(87)90059-5

[b41-grsb-2008-213] Nagel M Tahinci E Symes K 2004 Guidance of mesoderm cell migration in the *Xenopus* gastrula requires PDGF signaling Development 131 2727 36 15128658 10.1242/dev.01141

[b42-grsb-2008-213] Nakahashi T Yamana K 1976 Biochemical and cytological examination of the examination of the initiation of ribosomal RNA synthesis during gastrulation of *Xenopus laevis* Dev. Growth Differ 18 329 39 10.1111/j.1440-169X.1976.00329.x 37281758

[b43-grsb-2008-213] Nakajima K Takahashi A Yaoita Y 2000 Structure, expression, and function of the *Xenopus laevis* caspase family J. Biol. Chem 275 10484 91 10744739 10.1074/jbc.275.14.10484

[b44-grsb-2008-213] Nakakura N Miura T Yamana K 1987 Synthesis of heterogeneous mRNA-like RNA and low-molecular-weight RNA before the midblastula transition in embryos of *Xenopus laevis* Dev. Biol 123 421 9 2443406 10.1016/0012-1606(87)90400-3

[b45-grsb-2008-213] Newmeyer DD Farschon DM Reed JC 1994 Cell-free apoptosis in *Xenopus* egg extracts: inhibition by Bcl-2 and requirement for an organelle fraction enriched in mitochondria Cell 97 353 64 7954801 10.1016/0092-8674(94)90203-8

[b46-grsb-2008-213] Newport J Kirschner M 1982 A major developmental transition in early *Xenopus* embryos: I. Characterization and timing of cellular changes at the midblastula stage Cell 30 675 86 6183003 10.1016/0092-8674(82)90272-0

[b47-grsb-2008-213] Ninomiya H Elinson RP Winklbauer R 2004 Antero-posterior tissue polarity links mesoderm convergent extension to axial patterning Nature 430 364 7 15254540 10.1038/nature02620

[b48-grsb-2008-213] Nishikawa A Kaiho M Yoshizato K 1989 Cell. death in the anuran tadpole tail: thyroid hormone induces keratinization and tail-specific growth inhibition of epidermal cells Dev. Biol 131 337 44 2463945 10.1016/s0012-1606(89)80007-7

[b49-grsb-2008-213] Pegg AE Recent advances in the biochemistry of polyamines in eukaryotes Biochem J 234 249 62 3087344 10.1042/bj2340249 PMC1146560

[b50-grsb-2008-213] Richter K Good PJ Dawid IB 1990 A developmentally regulated, nervous system-specific gene in *Xenopus* encodes a putative RNA-binding protein New Biologist 2 556 65 1708282

[b51-grsb-2008-213] Richter JD Wasserman WJ Smith LD 1982 The mechanism for increased protein synthesis during *Xenopus* oocyte maturation Dev. Biol 89 159 67 7033020 10.1016/0012-1606(82)90304-9

[b52-grsb-2008-213] Salvesen GS Dixit VM 1997 Caspases: Intracellular signaling by proteolysis Cell 91 443 6 9390553 10.1016/s0092-8674(00)80430-4

[b53-grsb-2008-213] Shi Y-B Wong J Puzianowska-Kuznicka M 1996 Thyroid hormone receptors: Mechanisms of transcriptional regulation and roles during frog development J. Biomed. Sci 3 307 18 11725112 10.1007/BF02257960

[b54-grsb-2008-213] Shibata M Shing J Yasuhiko Y 1998 Overexpression of S-adenosylmethionine decarboxylase (SAMDC) in early *Xenopus* embryos induces cell dissociation and inhibits transition from the blastula to gastrula stage Int. J. Dev. Biol 42 675 86 9712522

[b55-grsb-2008-213] Shinga J Itoh M Shiokawa K Taira S Taira M 2001 Early patterning of the prospective midbrain-hindbrain boundary by the HES-related gene *XHR1* in *Xenopus* embryos Mech. Dev 109 225 39 11731236 10.1016/s0925-4773(01)00528-7

[b56-grsb-2008-213] Shinga J Kashiwagi K Tashiro K 1996 Maternal and zygotic expression of mRNA for S-adenosylmethionine decarboxylase and its relevance to the unique polyamine composition in *Xenopus* oocytes and embryos Biochim. Biophys. Acta 1308 31 40 8765748 10.1016/0167-4781(96)00020-6

[b57-grsb-2008-213] Shiokawa K Kai M Higo T 2000 Maternal program of apoptosis activated shortly after midblastula transition by overexpression of S-adenosylmethionine decarboxylase in *Xenopus* early embryos Comp. Biochem. Physiol. B 126 149 55 10874162 10.1016/s0305-0491(00)00193-0

[b58-grsb-2008-213] Shiokawa K Kurashima R Shinga J 1994 Temporal control of gene expression from endogenous and exogenously-introduced DNAs in early embryogenesis of *Xenopus laevis* Int. J. Dev. Biol 38 249 55 7526881

[b59-grsb-2008-213] Shiokawa K Misumi Y Tashiro K 1989 Changes in the patterns of RNA synthesis in early embryogenesis of *Xenopus laevis* Cell. Differ. Dev 28 17 25 2478271 10.1016/0922-3371(89)90019-1

[b60-grsb-2008-213] Shiokawa K Misumi Y Yamana K 1981a Demonstration of rRNA synthesis in pre-gastrular embryos of *Xenopus laevis* Develop. Growth Differ 23 579 87 10.1111/j.1440-169X.1981.00579.x 37281811

[b61-grsb-2008-213] Shiokawa K Tashiro K Misumi Y 1981b Non-coordinated synthesis of RNA’s in pre-gastrular embryos of *Xenopus laevis* Develop. Growth Differ 23 589 97 10.1111/j.1440-169X.1981.00589.x 37281934

[b62-grsb-2008-213] Shiokawa K Yamana K 1965 Demonstration of “polyphosphate” and its possible role in RNA synthesis during early development of Rana japonica embryos Exptl. Cell. Res 38 180 6 14281196 10.1016/0014-4827(65)90439-8

[b63-grsb-2008-213] Shiokawa K Yamana K 1967 Pattern of RNA synthesis in isolated cells of *Xenopus* laevis embryos Devlop. Biol 16 368 88 10.1016/0012-1606(67)90048-6 5621693

[b64-grsb-2008-213] Shiokawa K Yamana K 1979 Differential initiation of rRNA gene activity in progenies of different blastomeres of early *Xenopus* embryos: evidence for regulated synthesis of rRNA Dev. Growth Differ 21 501 7 10.1111/j.1440-169X.1979.00501.x 37281112

[b65-grsb-2008-213] Shiokawa K Yamana K Fu Y Atsuchi Y Hosokawa K 1990 Expression of exogenously introduced bacterial chloramphenicol acetyltransferase gene in *Xenopus laevis* embryos before the mid-blastula transition Roux’s Arch. dev Biol 198 322 9 10.1007/BF00383770 28305411

[b66-grsb-2008-213] Shiokawa K Takayama E Higo T 2005 Occurrence of pre-MBT synthesis of caspase-8 mRNA and activation of caspase-8 prior to execution of SAMDC (S-adenosylmethionine decarboxylase)-induced, but not p53-induced, apoptosis in *Xenopus* late blastulae Biochem. Biophys. Res. Commun 336 682 91 16143307 10.1016/j.bbrc.2005.08.144

[b67-grsb-2008-213] Shiratsuchi A Nakanishi Y 1999 Phosphatidyl serine-mediated phagocytosis of anticancer drug-treated cells by macrophages J. Biochem 126 1101 6 10578062 10.1093/oxfordjournals.jbchem.a022555

[b68-grsb-2008-213] Shook D Majer C Keller R 2004 Pattern and morphogenesis of presumptive superficial mesoderm in Two closely related species, *Xenopus laevis* and *Xenopus tropicalis* Dev. Biol 270 163 85 15136148 10.1016/j.ydbio.2004.02.021

[b69-grsb-2008-213] Sible JC Anderson JA Lewelly AL 1997 Zygotic transcription is required to block a maternal program of apoptosis in *Xenopus* embryos Dev. Biol 189 335 46 9299125 10.1006/dbio.1997.8683

[b70-grsb-2008-213] Signoret J Lefresne J 1973 Contribution à l’étude de la segmentation de l’oeuf d’axolotl. II. Influence de modifications du noyau et du cytoplasme surles modalités de la segmentation Ann. Embryol. Morphol 6 299 307

[b71-grsb-2008-213] Slee EA Harte MT Kluck RM 1999 Ordering the cytochrome c-initiated caspase cascade: hierarchial activation of caspase-2, -3, -6, -7, -8, and -10 in a caspase-9 dependent manner J. Cell. Biol 144 281 92 9922454 10.1083/jcb.144.2.281 PMC2132895

[b72-grsb-2008-213] Stack JH Newport JW 1997 Developmentally regulated activation of apoptosis early in *Xenopus* gastrulation results in cyclin A degradation during interphase of the cell cycle Development 124 3185 95 9272959 10.1242/dev.124.16.3185

[b73-grsb-2008-213] Stancheva I Hensey C Meehan RR 2001 Loss of the maintenance methyltransferase, xDnmt1, induces apoptosis in *Xenopus* embryos EMBO J 20 1963 73 11296229 10.1093/emboj/20.8.1963 PMC125419

[b74-grsb-2008-213] Takayama E Higo T Kai M 2004 Involvement of caspase-9 in execution of the maternal program of apoptosis in *Xenopus* late blastulae overexpressed with S-adenosylmethionine decdarboxylase Biochem. Biophys. Res. Commun 325 1367 75 15555578 10.1016/j.bbrc.2004.10.179

[b75-grsb-2008-213] Tata JR Kawahara A Baker BS 1991 Prolactin inhibits both thyroid hormone-induced morphogenesis and cell death in cultured amphibian larval tissues Dev. Biol 146 72 80 2060712 10.1016/0012-1606(91)90447-b

[b76-grsb-2008-213] Tchang F Gusse M Soussi T 1993 Stabilization and expression of high levels of p53 during early development in *Xenopus laevis* Dev. Biol 159 163 72 7689990 10.1006/dbio.1993.1230

[b77-grsb-2008-213] Thornberry NA 1997 The caspase family of cysteine proteases Br. Med. Bull 53 478 90 9374032 10.1093/oxfordjournals.bmb.a011625

[b78-grsb-2008-213] Wakiyama M Saigoh M Shiokawa K 1995 mRNA encoding the translation initiation factor eIF-4E is expressed early in *Xenopus* embryogenesis FEBS Lett 360 191 3 7875328 10.1016/0014-5793(95)00081-j

[b79-grsb-2008-213] Wallingford JB Seufert DW Virta VC 1997 p53 activity is essential for normal development in *Xenopus* Curr. Biol 7 747 57 9368757 10.1016/s0960-9822(06)00333-2

[b80-grsb-2008-213] Wang Z Brown DD 1993 Thyroid hormone-induced gene expression program for amphibian tail resorption J. Biol. Chem 268 16270 8 8344914

[b81-grsb-2008-213] Wroble BN Sible JC 2005 Chk2/Cds1 protein kinase blocks apoptosis during early development of *Xenopus laevis* Dev. Dyn 233 1359 65 15937936 10.1002/dvdy.20449

[b82-grsb-2008-213] Woodland HR 1974 Changes in the polysome content of developing *Xenopus* laevis embryos Dev. Biol 40 90 101 4472028 10.1016/0012-1606(74)90111-0

[b83-grsb-2008-213] Yang J Liu X Bhalla K 1997 Prevention of apoptosis by Bcl-2: release of cytochrome c from mitochondria blocked Science 275 1129 32 9027314 10.1126/science.275.5303.1129

[b84-grsb-2008-213] Yang J Tan C Darken RS 2002 β-Catenin/Tcf regulated transcription prior to the midblastula transition Development 129 5743 52 12421713 10.1242/dev.00150

[b85-grsb-2008-213] Yaoita Y Nakajima K 1997 Induction of apoptosis and CPP32 expression by thyroid hormone in a myoblastic cell line derived from tadpole tail J. Biol. Chem 272 5122 7 9030578 10.1074/jbc.272.8.5122

[b86-grsb-2008-213] Yasuda GK Schubiger G 1992 Temporal regulation in the early embryo: Is MBT too good to be true Trends Genet 8 124 7 1631954 10.1016/0168-9525(92)90369-F

